# Supplementary material for: Chromosome-level genome assemblies of the malaria vectors Anopheles coluzzii and Anopheles arabiensis
Source: Gigascience. 2021 Mar 15;10(3):giab017. doi: 10.1093/gigascience/giab017 (PMC7957348; doi:10.1093/gigascience/giab017)
Supplement: giab017_Supplemental_Files [file giab017_supplemental_files.zip › Additional file 12.docx]

**
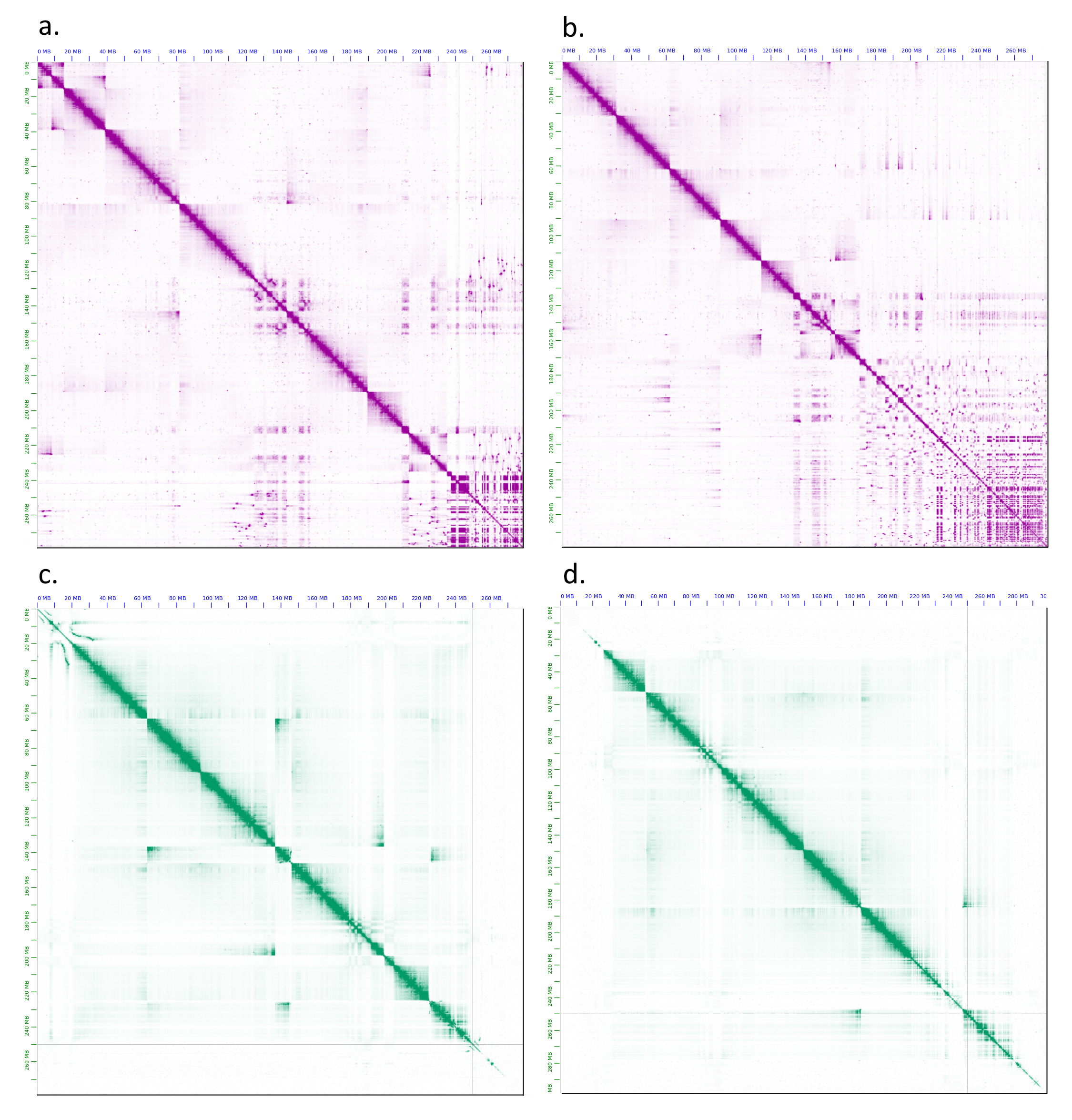
**

**Additional file 12.** The initial heat maps of Hi-C contact information for the *An. arabiensis* genome assemblies obtained by **(a)** SALSA 2 from the Canu contig assembly, **(b)** SALSA 2 from the Canu unitig assembly, **(c)** 3D-DNA from the Canu contig assembly, and **(d)** 3D-DNA from the Canu unitig assembly. The heat maps are produced by JBAT.
