# Supplementary material for: Chromosome-level genome assemblies of the malaria vectors Anopheles coluzzii and Anopheles arabiensis
Source: Gigascience. 2021 Mar 15;10(3):giab017. doi: 10.1093/gigascience/giab017 (PMC7957348; doi:10.1093/gigascience/giab017)
Supplement: giab017_Supplemental_Files [file giab017_supplemental_files.zip › Additional file 13.docx]

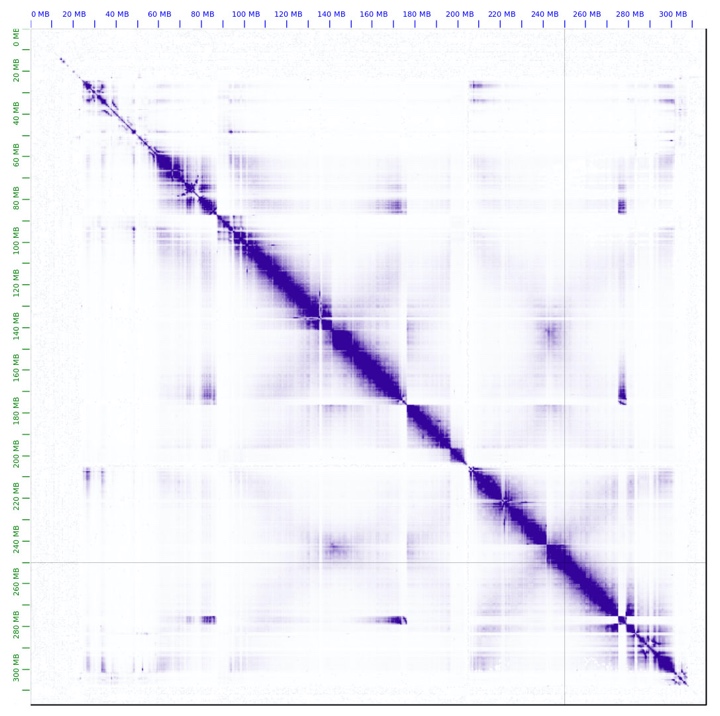


**Additional file 13.** The Hi-C contact heat map for the 3D-DNA scaffolds of the *An. coluzzii* assembly before manual correction. The heat map is produced by JBAT.
