# Supplementary material for: Chromosome-level genome assemblies of the malaria vectors Anopheles coluzzii and Anopheles arabiensis
Source: Gigascience. 2021 Mar 15;10(3):giab017. doi: 10.1093/gigascience/giab017 (PMC7957348; doi:10.1093/gigascience/giab017)
Supplement: giab017_Supplemental_Files [file giab017_supplemental_files.zip › Additional file 14.docx]

**
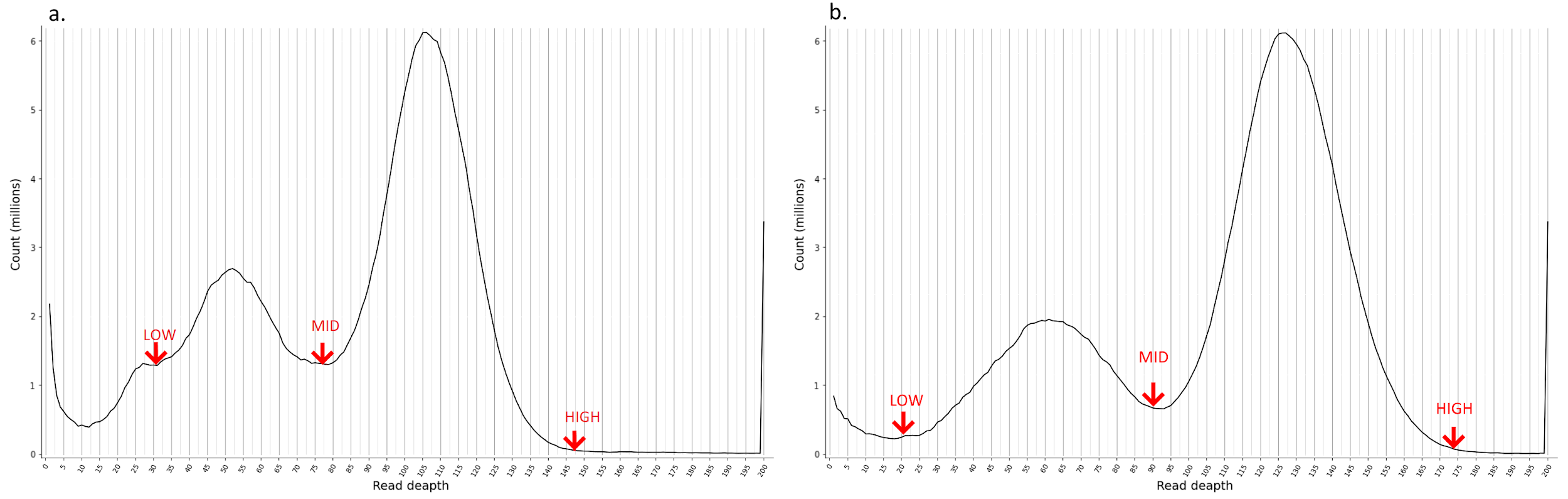
**

**Additional file 14.** The read depth histogram obtained by Purge Haplo for the *An. coluzzii* **(a)** and *An. arabiensis* **(b)** assemblies. The cut-offs were manually selected (red arrows in the histograms): 30, 78, and 132 for *An. сoluzzii* and 25, 93, and 160 for *An. arabiensis*.
