# Supplementary material for: Chromosome-level genome assemblies of the malaria vectors Anopheles coluzzii and Anopheles arabiensis
Source: Gigascience. 2021 Mar 15;10(3):giab017. doi: 10.1093/gigascience/giab017 (PMC7957348; doi:10.1093/gigascience/giab017)
Supplement: giab017_Supplemental_Files [file giab017_supplemental_files.zip › Additional file 16.docx]

***
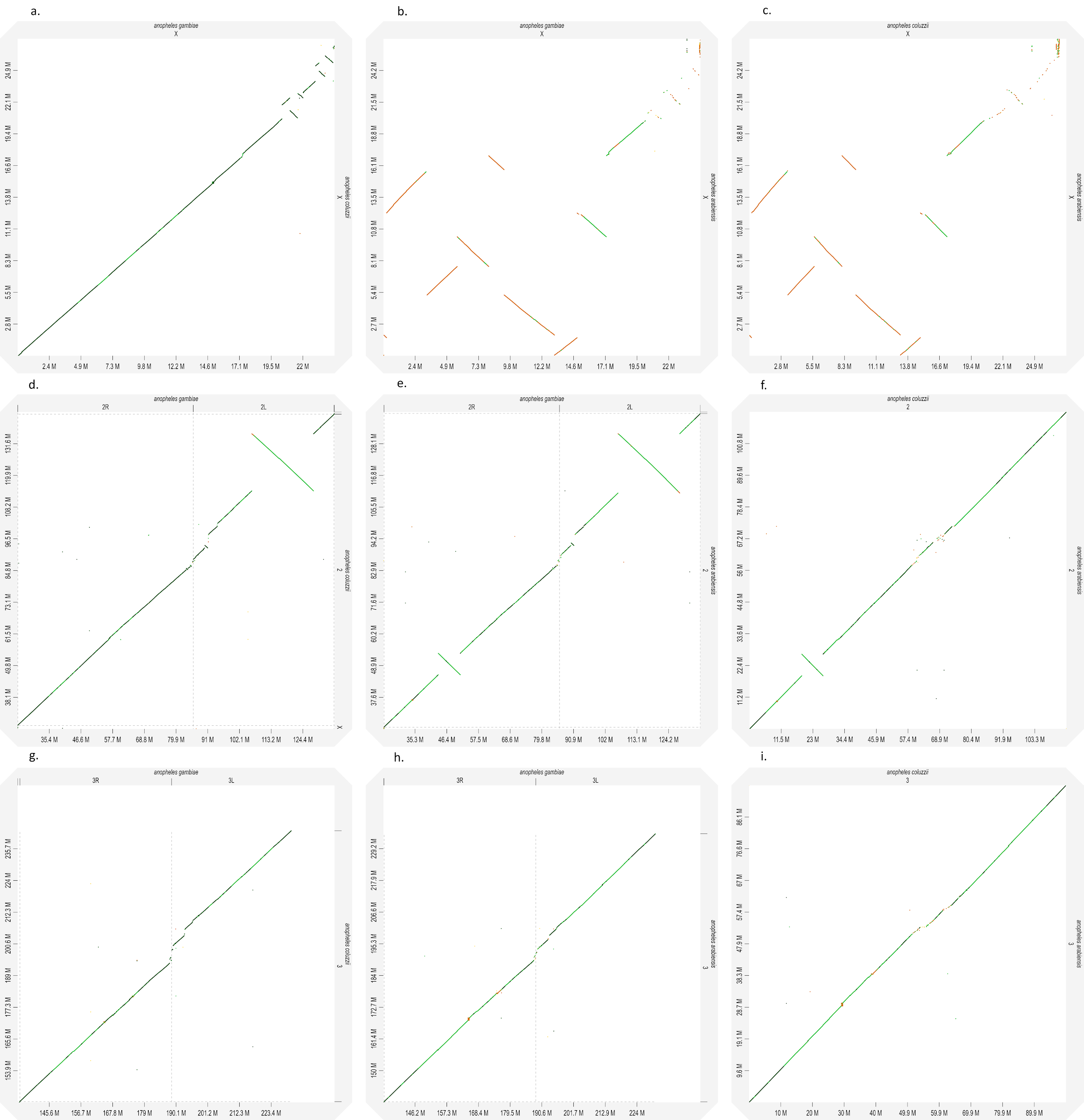
***

**Additional file 16.** Whole-genome pairwise alignment dot-plots (produced by D-Genies v1.2.0) between the scaffolds corresponding to the chromosomes X **(a, b, c)**, 2 **(d, e, f)**, 3 **(g, h, i)**. Alignments between the *An. gambiae* and *An. coluzzii* scaffolds*,* the *An. gambiae* and *An. arabiensis* scaffolds, the *An. coluzzii* and *An. arabiensis* scaffolds are show.
