# Supplementary material for: Chromosome-level genome assemblies of the malaria vectors Anopheles coluzzii and Anopheles arabiensis
Source: Gigascience. 2021 Mar 15;10(3):giab017. doi: 10.1093/gigascience/giab017 (PMC7957348; doi:10.1093/gigascience/giab017)
Supplement: giab017_Supplemental_Files [file giab017_supplemental_files.zip › Additional file 20.docx]

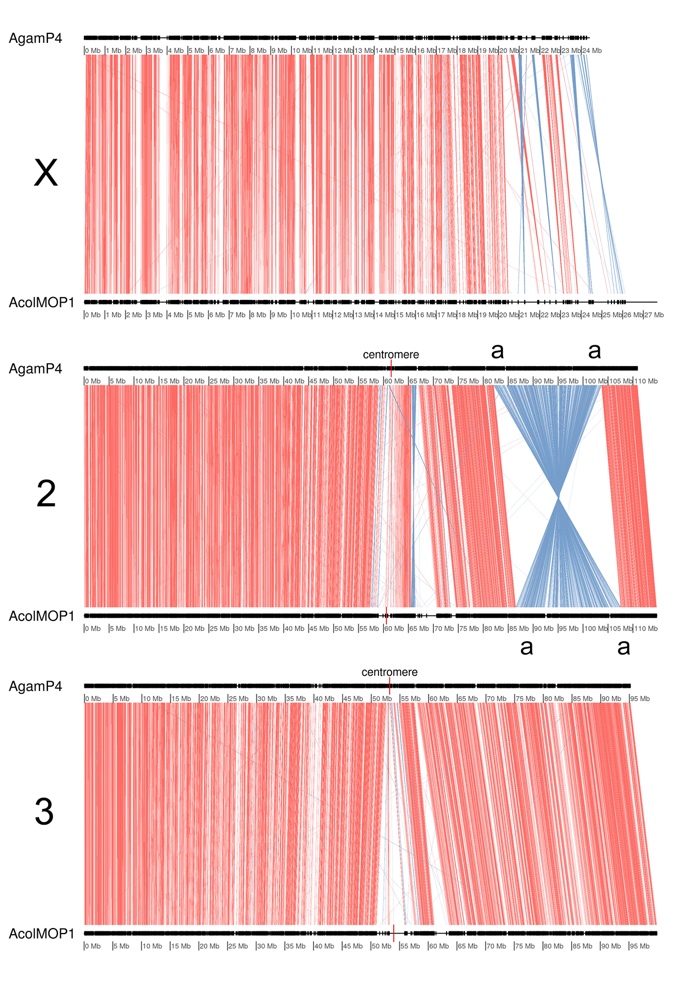

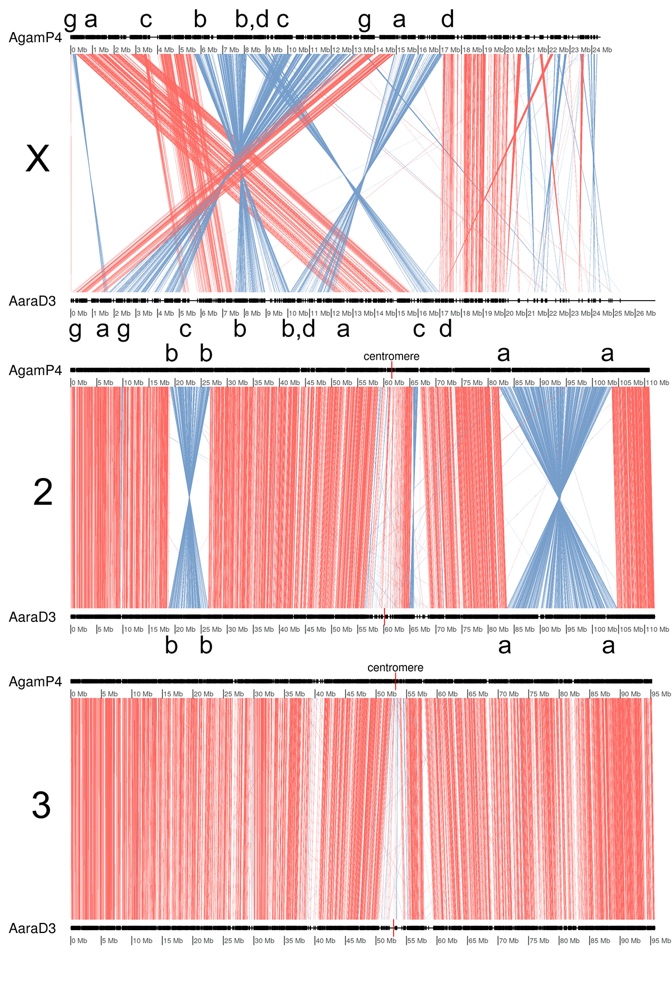

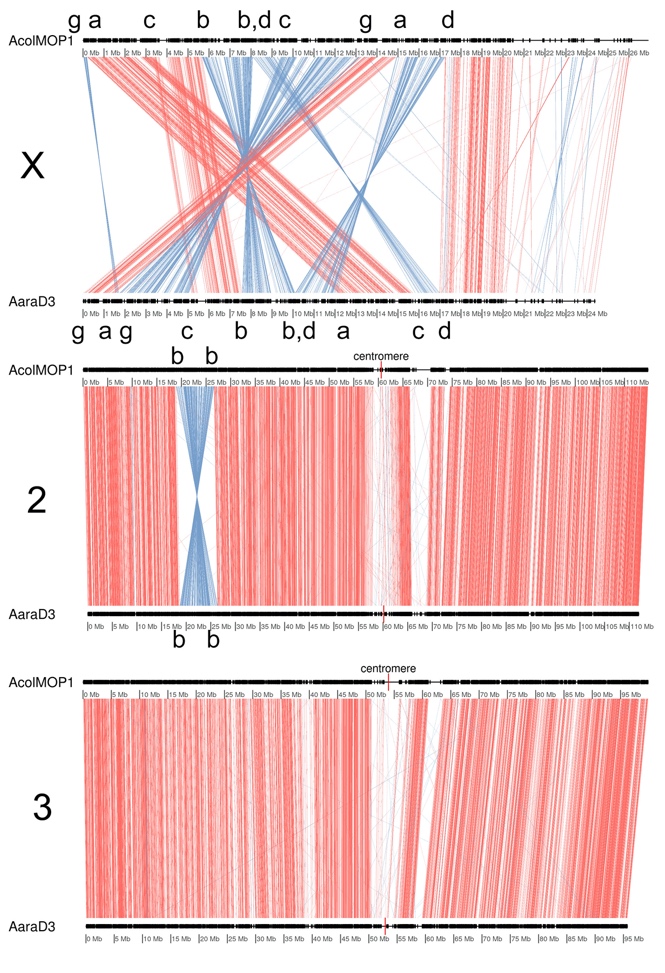


**Additional file 20.** Whole-genome pairwise alignments produced by genoPlotR between chromosomes of *An. arabiensis, An. coluzzii, and An. gambiae* Left panel: AgamP4 and AcolMOP1. Middle panel: AgamP4 and AaraD3. Right panel: AcolMOP1 and AaraD3.

The inversion breakpoints are shown with small letters.
