# Supplementary material for: Chromosome-level genome assemblies of the malaria vectors Anopheles coluzzii and Anopheles arabiensis
Source: Gigascience. 2021 Mar 15;10(3):giab017. doi: 10.1093/gigascience/giab017 (PMC7957348; doi:10.1093/gigascience/giab017)
Supplement: giab017_Supplemental_Files [file giab017_supplemental_files.zip › Additional file 21.docx]

**
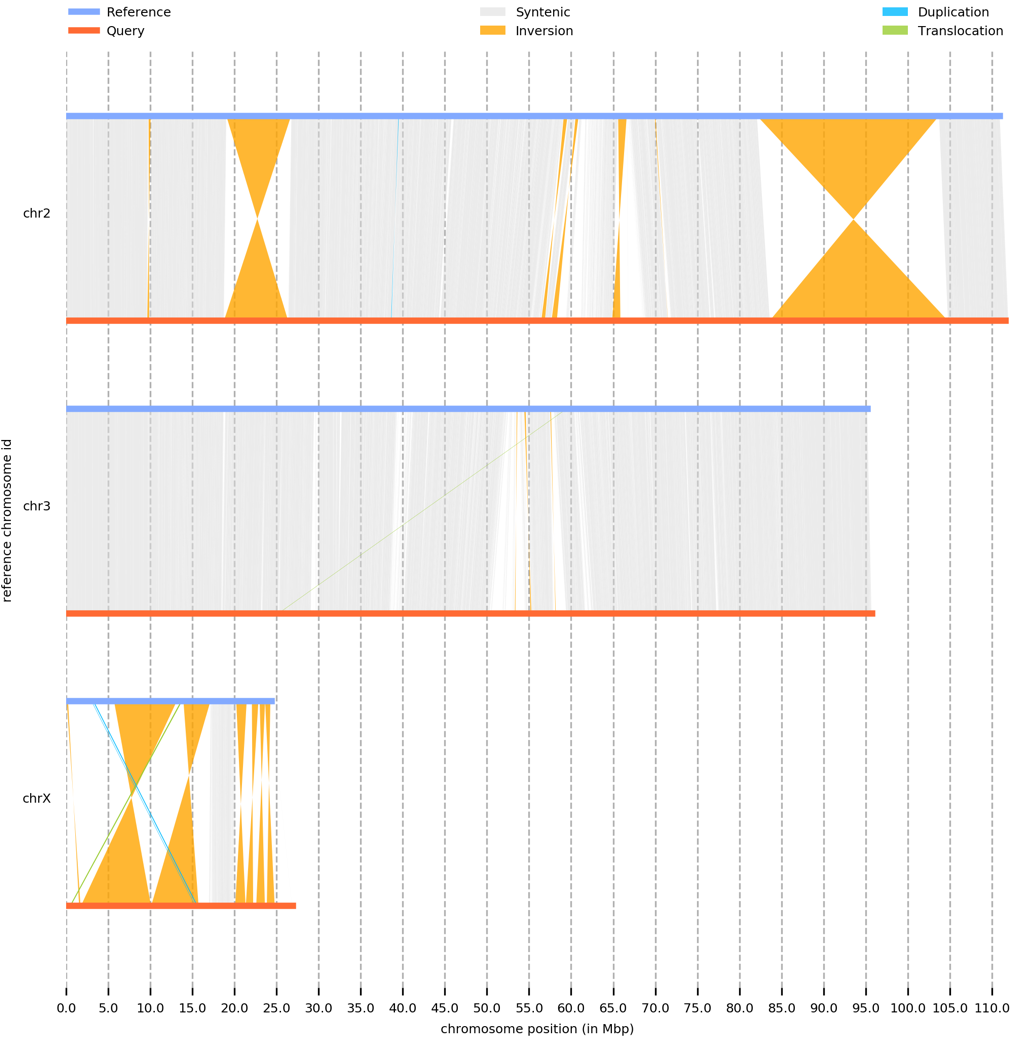

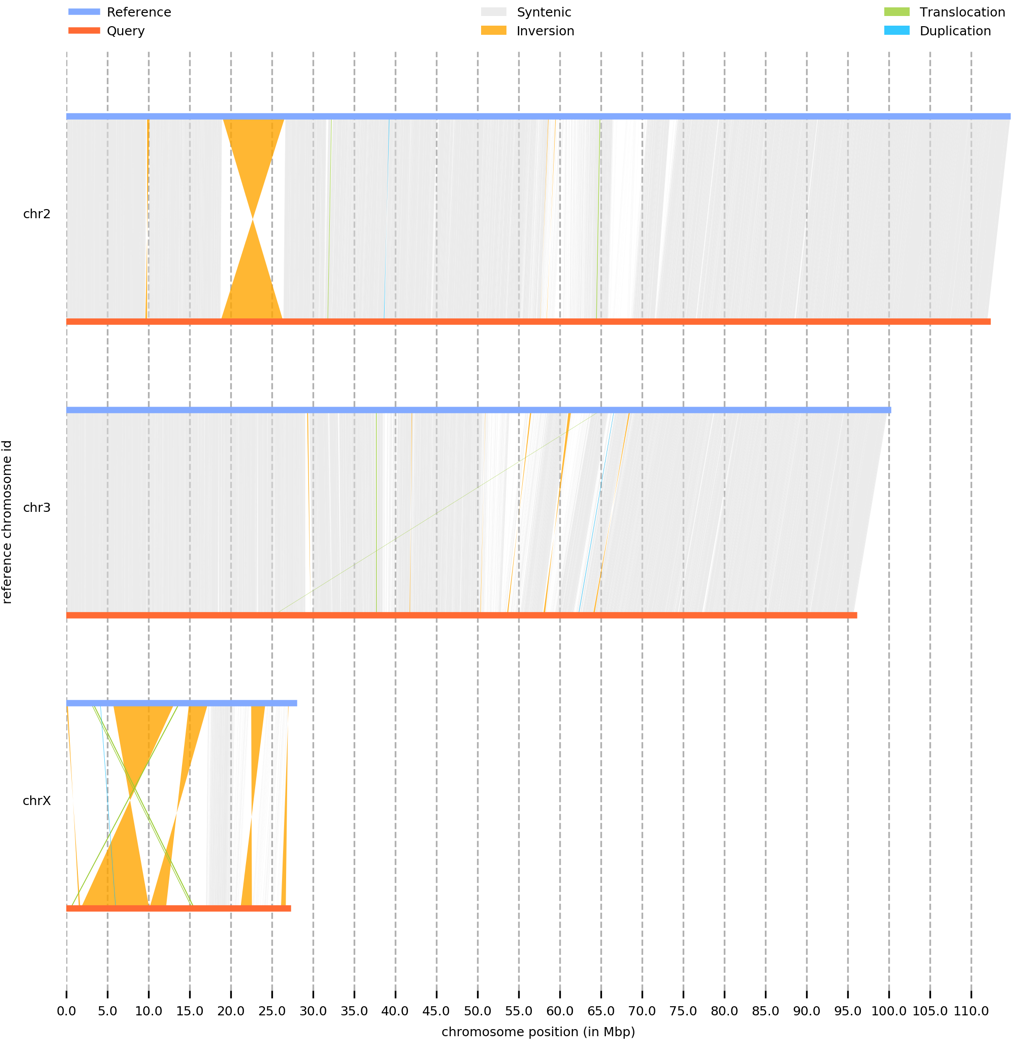
**

**Additional file 21.** Whole-genome pairwise alignments produced by SyRi of *An. arabiensis* chromosomes (query) to *An. gambiae* (reference)*,* and *An. coluzzii* (reference) chromosomes. Left panel: AgamP4 and AaraD3. Right panel: AcolMOP1 and AaraD3.
