# Supplementary material for: Chromosome-level genome assemblies of the malaria vectors Anopheles coluzzii and Anopheles arabiensis
Source: Gigascience. 2021 Mar 15;10(3):giab017. doi: 10.1093/gigascience/giab017 (PMC7957348; doi:10.1093/gigascience/giab017)
Supplement: giab017_Supplemental_Files [file giab017_supplemental_files.zip › Additional file 24.docx]

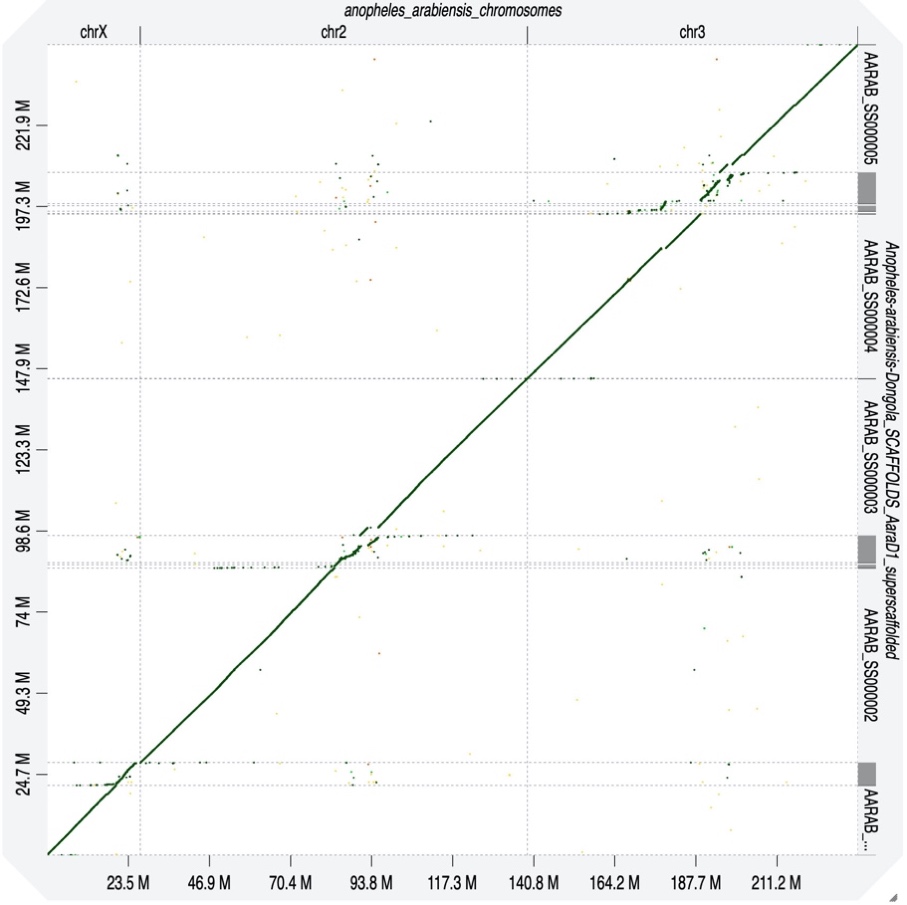


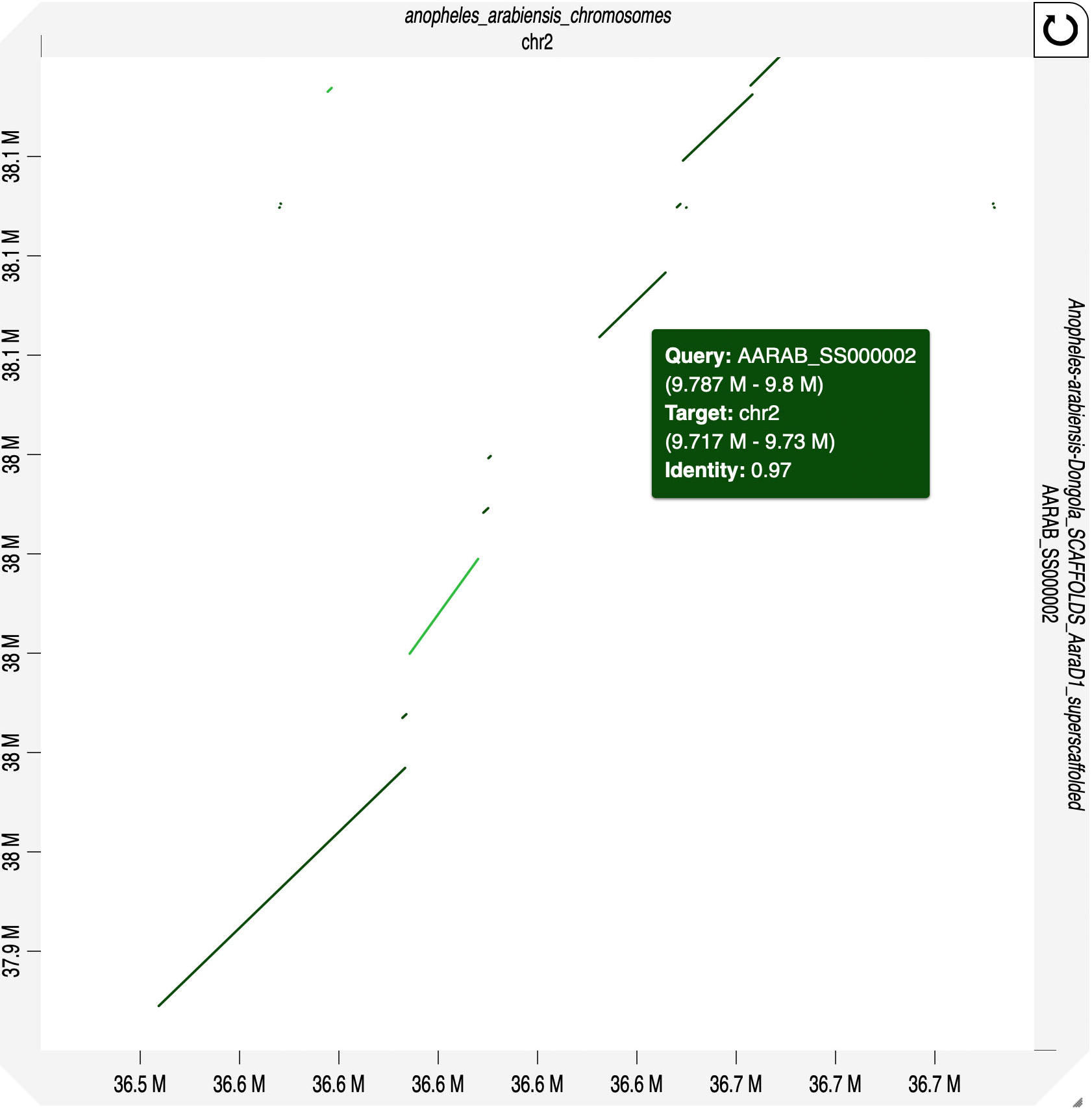


**Additional file 24.** Pairwise dot-plot alignment between the AaraD3 and AaraD2 (AaraD1 superscaffolded) assemblies produced by D-Genies v1.2.0. Top panel: Whole-genome pairwise alignment. Bottom panel: Collinearity in the alignment of the region with the new 2R microinversion.
