# Supplementary material for: Chromosome-level genome assemblies of the malaria vectors Anopheles coluzzii and Anopheles arabiensis
Source: Gigascience. 2021 Mar 15;10(3):giab017. doi: 10.1093/gigascience/giab017 (PMC7957348; doi:10.1093/gigascience/giab017)
Supplement: giab017_Supplemental_Files [file giab017_supplemental_files.zip › Additional file 25.docx]

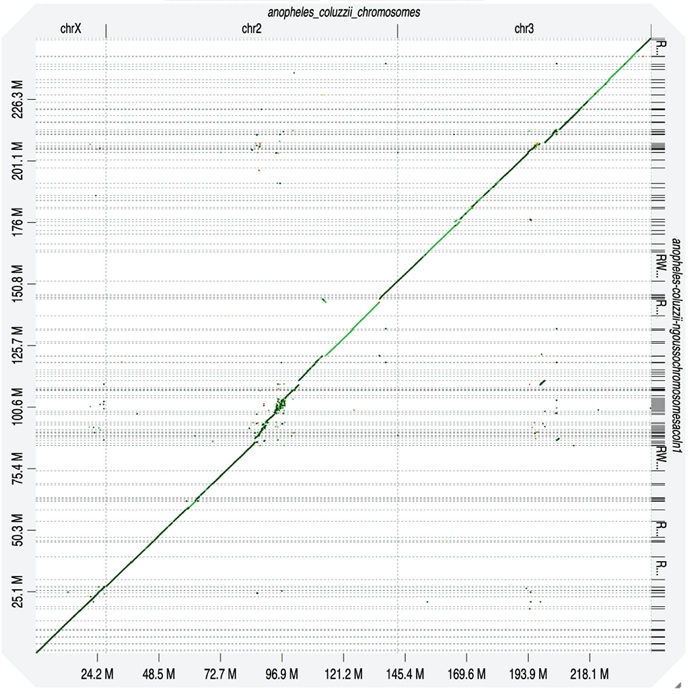

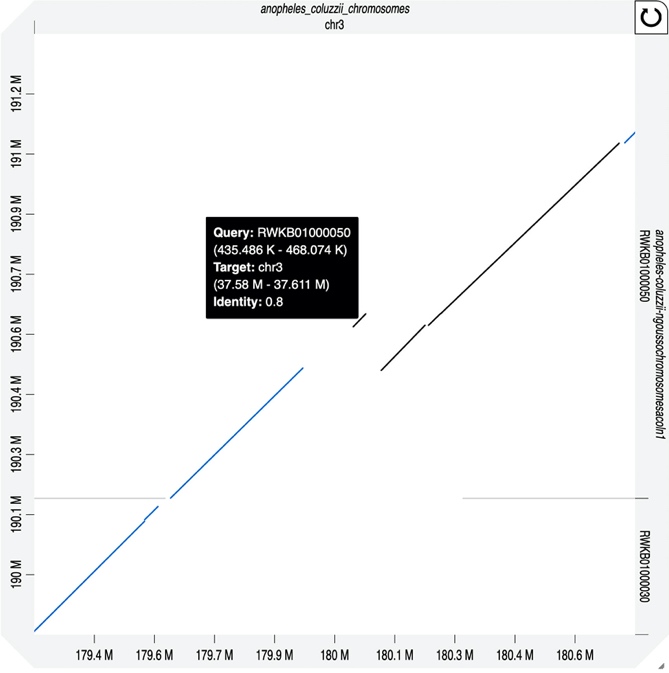

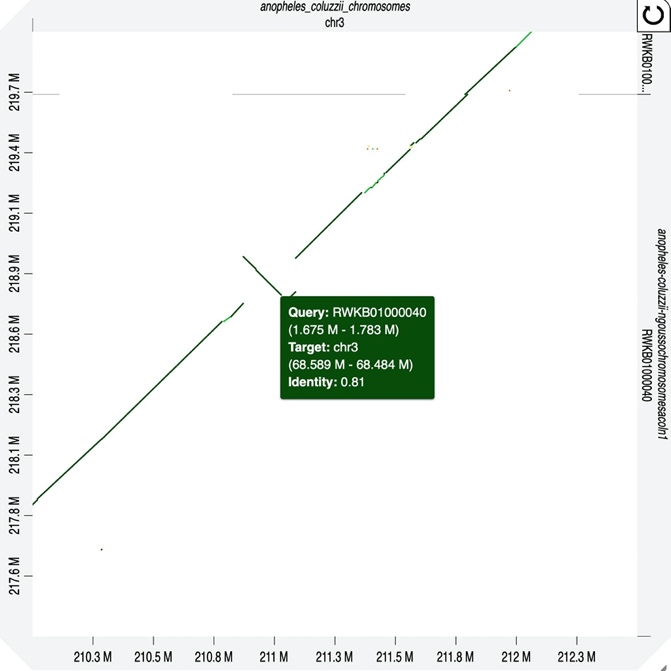


**Additional file 25.** Pairwise dot-plot alignment between the AcolMOP1 and reference-guided scaffolded AcolN1 assemblies produced by D-Genies v1.2.0. Left panel: Whole-genome pairwise alignment. Middle panel: The alignment of the region with the new 3R translocation. Right panel: The alignment of the region with the new 3L microinversion.
