# Supplementary material for: Chromosome-level genome assemblies of the malaria vectors Anopheles coluzzii and Anopheles arabiensis
Source: Gigascience. 2021 Mar 15;10(3):giab017. doi: 10.1093/gigascience/giab017 (PMC7957348; doi:10.1093/gigascience/giab017)
Supplement: giab017_Supplemental_Files [file giab017_supplemental_files.zip › Additional file 27.docx]

**
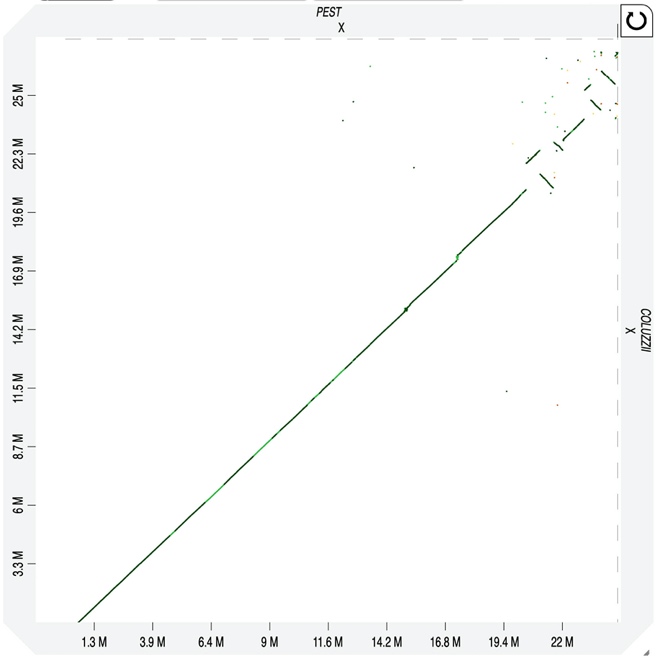

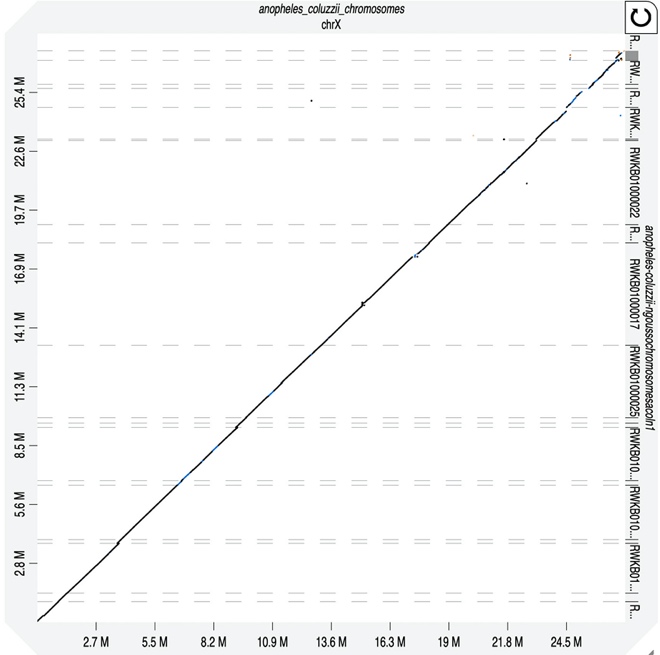

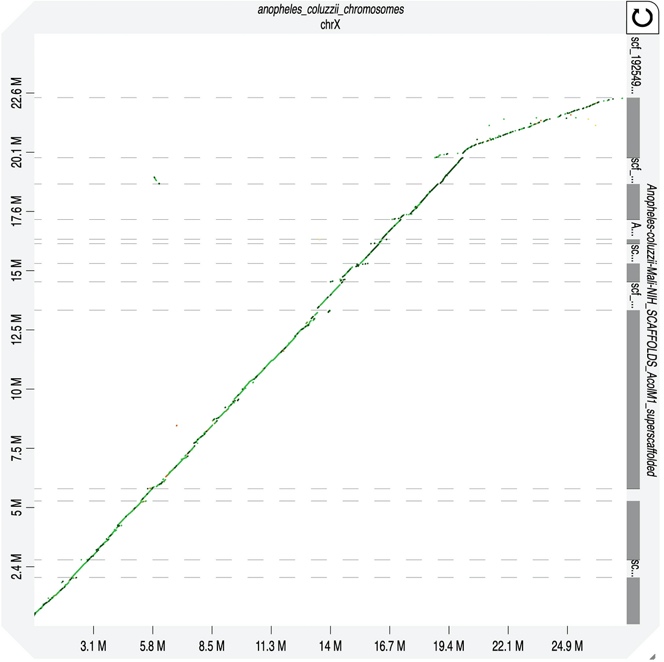
**

**Additional file 27.** Pairwise dot-plot alignment between the X chromosomes produced by D-Genies v1.2.0. Left panel: AcolMOP1 and AgamP4. Middle panel: AcolMOP1 and AcolN1. Right panel: AcolMOP1 and AcolM2.
