# Supplementary material for: Chromosome-level genome assemblies of the malaria vectors Anopheles coluzzii and Anopheles arabiensis
Source: Gigascience. 2021 Mar 15;10(3):giab017. doi: 10.1093/gigascience/giab017 (PMC7957348; doi:10.1093/gigascience/giab017)
Supplement: giab017_Supplemental_Files [file giab017_supplemental_files.zip › Additional file 5.docx]

**
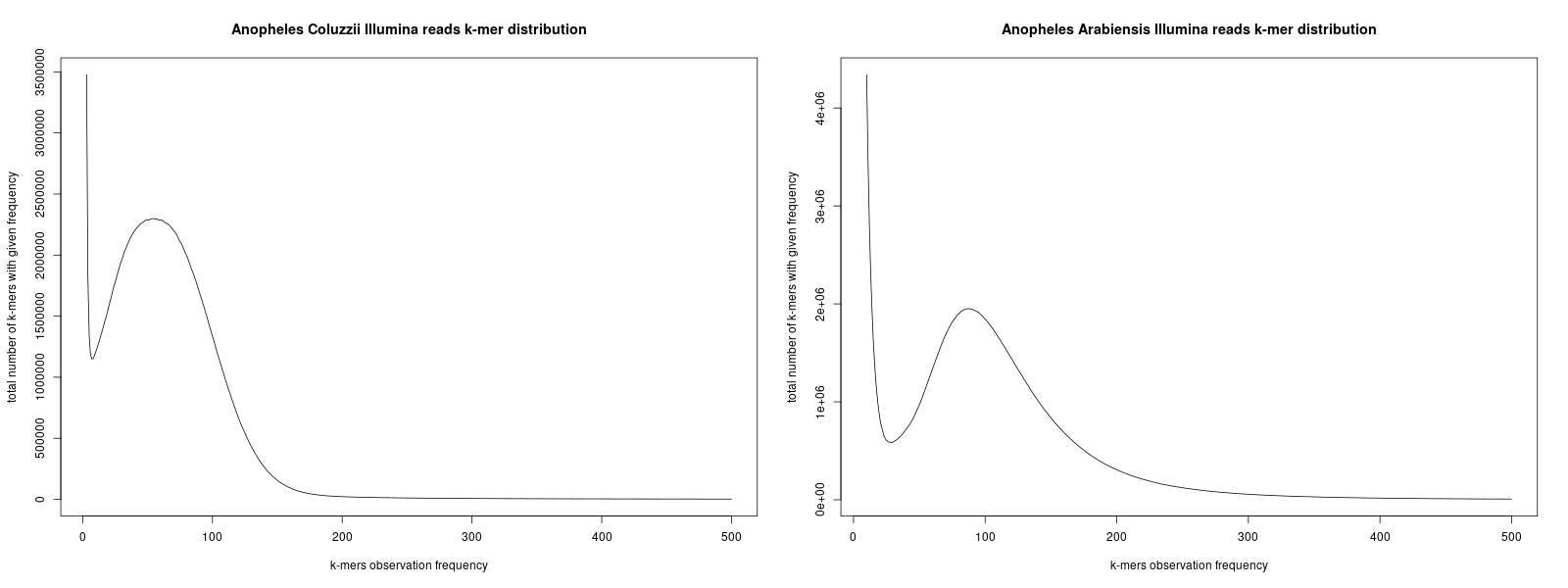
**

**Additional file 5.** Distribution of 19-mers for *An. coluzzii* (left panel) and *An. arabiensis* (right panel) computed by Jellyfish.
