# Supplementary material for: Chromosome-level genome assemblies of the malaria vectors Anopheles coluzzii and Anopheles arabiensis
Source: Gigascience. 2021 Mar 15;10(3):giab017. doi: 10.1093/gigascience/giab017 (PMC7957348; doi:10.1093/gigascience/giab017)
Supplement: giab017_Response_to_Reviewer_Comments_Original_Submission [file giab017_response_to_reviewer_comments_original_submission.pdf]

Author's Response To Reviewer Comments

Close

Reviewer reports:
Reviewer #1: This is a comprehensive description of two new genome assemblies for the important vector species Anopheles coluzzii and Anopheles arabiensis. I have no doubt that these assemblies will be useful to the field. The A. gambiae species complex is unusually diverse and shows complicated patterns of introgression, chromosomal rearrangements, and barriers to gene flow. These Nanopore/Hi-C assemblies will clarify many of these diversity patterns. It is likely that many other third-generation sequencing assemblies will soon be available for Anopheles (e.g. the authors mention the PacBio assembly from A. coluzzii Ngousso), but few have been released yet, and in any case the diversity of these species means that the genomes will not be redundant. Here, the authors have thoroughly assessed the data and compared the genomes to each other and to the A. gambiae genome which is the standard reference for this genus. The methods are robust and well documented.

Response: Thank you for this assessment.

"Genomics analyses" should be "Genomic analyses"
Response: Corrected

"scaffold N50s are 99.9 and 95.7" The units here are clearly Mbp but still this should be specifically stated.
Response: Corrected

Reviewer #2: The authors present us chromosomal level genome assemblies of two malaria vector species in Anopheles. The materials and methods were well described and the assembly results look promising, and I believe the two high quality genomes will be valuable genomics sources for further scientific research. The authors tried multiple methods to validate the genome assemblies and the related findings, but I noticed that the genome assemblies could include some obvious errors according to the Hi-C heat maps. For example, there is too much proportion debris that cannot be assigned to their corresponding chromosomes, ca. 15% contigs cannot be clustered into their corresponding chromosome locations. Plus, I also noticed several obvious mis-assemblies (or mis-clusters) for the assemblies of AcolMOP1 - there are some regions along the diagonal that have their Hi-C signals placed at wrong positions. However, I admit that the authors, via applying multiple widely-used genome assembly tools, have already tried their best to obtain the most reliable assemblies for their data. This could be the best results they can achieve uptonow due to the limitations of sequencing technologies and computational methods. As a result, I recommend its publication after addressing the issues as follows:

1. Although the authors have already tried multiple assembly tools for their ONT sequences, I can still observe several obvious mis-assemblies from the Hi-C heat maps. I would recommend two assembly tools that are designed for the ONT long noisy reads, which, as far I know, can perform better than those tools applied in the current work.

https://github.com/xiaochuanle/NECAT
https://github.com/Nextomics/NextDenovo

However, I am also aware that the performance of bioinformatics tools varies a lot owing to the variety of genomes inherited from biodiversity. Therefore, the authors do not have to apply a full genome analysis pipeline for the two software in case they cannot produce better assembly results compared to the ones you have.
Response: Thank you for your assessment and suggestions. We believe that by trying multiple assembly tools our pipeline produced the best assemblies possible using the sequencing data for these mosquito species. As for the observed several “mis-assemblies” from the Hi-C heat maps, we are not completely sure what regions are in question. If they are located within the chromosomal arms, these are inner parts of the contigs assembled by CANU. The off-diagonal signals on the A. coluzzii Hi-C map are caused

by the normalization and visualization algorithm of JBAT. We cannot precisely determine the real cause of these transchromosomal 2R-3R signals. They can be either misassemblies by CANU or technical problems with the Hi-C reads alignment in repeat regions. In any case, these signals occupy very small regions on 2R. If they are located in the debris region, then they indeed can be mis-assemblies but they are not part of the chromosome-level assembly. The debris region consists of haplotigs, assembly artifacts, and small genome contigs that have no Hi-C signal to be assigned to any scaffold. Most of these signals are caused by haplotigs. To address this issue, we provided a new figure 2 with lines separating chromosome arms. This new figure was obtained after removing debris regions and unlocalized scaffolds (X pericentromeric, autosomal pericentromeric, and Y unlocalized). These regions lack the Hi-C signal and, therefore, they were scaffolded with additional methods during validation (CQ analysis, satellite mapping, mapping of A. gambiae genes).

2. The authors may want to put some of the additional files, especially those figures and small tables, into one single additional file and name them as supplementary figures and tables to improve readability.
Response: We have decided to keep the current format since it is easier to go to a specific supplementary figure or table just by clicking on the hyperlink in the text.

3. The authors may want to depict their results more carefully. For example, (1) the median read length was 3.8 kbp and 2.3 kbp for An. coluzzii and An. arabiensis, respectively, according to additional file 1, rather than 4 kbp and 2.2 kbp described in your main text; (2) Although CANU generated the third highest number of mis-assemblies, it also has a longer alignment length compared to the assemblies produced by WTBG2 et al. The long alignments could have contributed to those more mis-assemblies, which needs additional explanations; (3) I cannot achieve the conclusion of single copy genomic regions of 204.1 Mbp from additional file 5 (Page 7); and some others I won't point them out one by one here.
Response:
(1) The median read length in the text was corrected according to additional file 1.
(2) In our main text, we focused on two metrics (NG50 and the number of misassemblies) since they are good representative measures that can be understood by a broad audience. We sought a trade-off between explaining our decisions for a broader audience and correctness of our explanations. However, we provided full QUAST reports after each stage of our pipeline for experts in genome assembly. So, they would be able to derive their own conclusions considering more parameters than just the two mentioned above.
We agree with the reviewer that the relationship between the number of misassemblies and alignment length exists and should be considered before deciding with which assembly to proceed further. While some assemblers tend to produce longer contigs (i.e., trying harder to resolve some complex regions), other assemblers take a more conservative approach and produce shorter contigs. Considering that scaffolding methods with Hi-C allow not only joining contigs but also correcting misassemblies, it is not 100% clear based on which metric an assembly should be chosen. Moreover, while it is natural to assume that longer alignment lengths tend to lead to a larger number of misassemblies, it may be not so obvious in our case since the reference genome is different from the assembled genomes. Thus, some “misassemblies” are not misassemblies at all.
Overall, we made our decisions based on all metrics in the QUAST report as well as some manual investigations of obtained assemblies. We believe that a lot of nuances about these metrics represent interest only for experts in the genome assembly community and may be omitted for representation purposes. We think that the NG50 metric has some weak connection with alignment length and can be used as a representative one.
(3) To estimate the genome size and size of single-copy genomic regions we used methodology described in the genome size estimation tutorial [1]. The figure in additional file 5 shows distributions of 19-mers from Illumina reads for each species. We separated a 19-mer distribution into three consecutive ranges that correspond to error sequences, single copy sequences (i.e., haploid peak), and repeat sequences. We estimated the average 19-mer single copy coverage by finding maximum in haploid peak. After that, we calculated the area under the curve for the whole distribution range except sequencing error range. For obtaining genome length, the obtained area was divided by the coverage calculated in the previous step. The length of the single copy sequences was calculated in the same manner but only for the single copy sequence range. The formulas for calculating respective lengths are given in the Methods. It is important to mention one key assumption that was made about our data for analyses described above. Illumina data for both species were obtained by sequencing colonies of mosquitoes (i.e., mix of different individual genomes). Therefore, we expect a high heterozygosity rate.

We will be glad to answer how we derived other lengths if any questions remain.

[1] https://bioinformatics.uconn.edu/genome-size-estimation-tutorial/

4. End-to-end genome assembly, do you mean Telomere-to-Telomere genome assembly.
Response: We have changed to “telomere-to-telomere.”

Close
