# Supplementary material for: Chromosome-level genome assemblies of the malaria vectors Anopheles coluzzii and Anopheles arabiensis
Source: Gigascience. 2021 Mar 15;10(3):giab017. doi: 10.1093/gigascience/giab017 (PMC7957348; doi:10.1093/gigascience/giab017)
Supplement: giab017_Reviewer_1_Report_Original_Submission — Jacob Tennessen -- 11/12/2020 Reviewed [file giab017_reviewer_1_report_original_submission.pdf]

Reviewer Report

Title: Chromosome-level genome assemblies of the malaria vectors Anopheles coluzzii and Anopheles arabiensis

Version: Original Submission **Date:** 11/12/2020

Reviewer name: Jacob Tennessen

Reviewer Comments to Author:

This is a comprehensive description of two new genome assemblies for the important vector species Anopheles coluzzii and Anopheles arabiensis. I have no doubt that these assemblies will be useful to the field. The A. gambiae species complex is unusually diverse and shows complicated patterns of introgression, chromosomal rearrangements, and barriers to gene flow. These Nanopore/Hi-C assemblies will clarify many of these diversity patterns. It is likely that many other third-generation sequencing assemblies will soon be available for Anopheles (e.g. the authors mention the PacBio assembly from A. coluzzii Ngousso), but few have been released yet, and in any case the diversity of these species means that the genomes will not be redundant. Here, the authors have thoroughly assessed the data and compared the genomes to each other and to the A. gambiae genome which is the standard reference for this genus. The methods are robust and well documented.
"Genomics analyses" should be "Genomic analyses"
"scaffold N50s are 99.9 and 95.7" The units here are clearly Mbp but still this should be specifically stated.

Level of Interest

Please indicate how interesting you found the manuscript: Choose an item.

Quality of Written English

Please indicate the quality of language in the manuscript: Choose an item.

Declaration of Competing Interests

Please complete a declaration of competing interests, considering the following questions:

- Have you in the past five years received reimbursements, fees, funding, or salary from an organisation that may in any way gain or lose financially from the publication of this manuscript, either now or in the future?
- Do you hold any stocks or shares in an organisation that may in any way gain or lose financially from the publication of this manuscript, either now or in the future?
- Do you hold or are you currently applying for any patents relating to the content of the manuscript?

- Have you received reimbursements, fees, funding, or salary from an organization that holds or has applied for patents relating to the content of the manuscript?
- Do you have any other financial competing interests?
- Do you have any non-financial competing interests in relation to this paper?

If you can answer no to all of the above, write 'I declare that I have no competing interests' below. If your reply is yes to any, please give details below.

I declare that I have no competing interests

I agree to the open peer review policy of the journal. I understand that my name will be included on my report to the authors and, if the manuscript is accepted for publication, my named report including any attachments I upload will be posted on the website along with the authors' responses. I agree for my report to be made available under an Open Access Creative Commons CC-BY license (http://creativecommons.org/licenses/by/4.0/). I understand that any comments which I do not wish to be included in my named report can be included as confidential comments to the editors, which will not be published.

Choose an item.

To further support our reviewers, we have joined with Publons, where you can gain additional credit to further highlight your hard work (see: https://publons.com/journal/530/gigascience). On publication of this paper, your review will be automatically added to Publons, you can then choose whether or not to claim your Publons credit. I understand this statement.

Yes Choose an item.
