# Supplementary material for: Chromosome-level genome assemblies of the malaria vectors Anopheles coluzzii and Anopheles arabiensis
Source: Gigascience. 2021 Mar 15;10(3):giab017. doi: 10.1093/gigascience/giab017 (PMC7957348; doi:10.1093/gigascience/giab017)
Supplement: giab017_Reviewer_2_Report_Original_Submission — Shanlin Liu -- 11/28/2020 Reviewed [file giab017_reviewer_2_report_original_submission.pdf]

Reviewer Report

Title: Chromosome-level genome assemblies of the malaria vectors Anopheles coluzzii and Anopheles arabiensis

Version: Original Submission **Date:** 11/28/2020

Reviewer name: Shanlin Liu

Reviewer Comments to Author:

The authors present us chromosomal level genome assemblies of two malaria vector species in Anopheles. The materials and methods were well described and the assembly results look promising, and I believe the two high quality genomes will be valuable genomics sources for further scientific research. The authors tried multiple methods to validate the genome assemblies and the related findings, but I noticed that the genome assemblies could include some obvious errors according to the Hi-C heat maps. For example, there is too much proportion debris that cannot be assigned to their corresponding chromosomes, ca. 15% contigs cannot be clustered into their corresponding chromosome locations. Plus, I also noticed several obvious mis-assemblies (or mis-clusters) for the assemblies of AcolMOP1 - there are some regions along the diagonal that have their Hi-C signals placed at wrong positions. However, I admit that the authors, via applying multiple widely-used genome assembly tools, have already tried their best to obtain the most reliable assemblies for their data. This could be the best results they can achieve uptonow due to the limitations of sequencing technologies and computational methods. As a result, I recommend its publication after addressing the issues as follows:

1. Although the authors have already tried multiple assembly tools for their ONT sequences, I can still observe several obvious mis-assemblies from the Hi-C heat maps. I would recommend two assembly tools that are designed for the ONT long noisy reads, which, as far I know, can perform better than those tools applied in the current work.
https://github.com/xiaochuanle/NECAT
https://github.com/Nextomics/NextDenovo
However, I am also aware that the performance of bioinformatics tools varies a lot owing to the variety of genomes inherited from biodiversity. Therefore, the authors do not have to apply a full genome analysis pipeline for the two software in case they cannot produce better assembly results compared to the ones you have.

2. The authors may want to put some of the additional files, especially those figures and small tables, into one single additional file and name them as supplementary figures and tables to improve readability.

3. The authors may want to depict their results more carefully. For example, (1) the median read length was 3.8 kbp and 2.3 kbp for An. coluzzii and An. arabiensis, respectively, according to additional file 1, rather than 4 kbp and 2.2 kbp described in your main text; (2) Although CANU generated the third highest number of mis-assemblies, it also has a longer alignment length compared to the assemblies produced by WTBG2 et al. The long alignments could have contributed to those more mis-assemblies, which needs additional explanations; (3) I cannot achieve the conclusion of single copy genomic regions

of 204.1 Mbp from additional file 5 (Page 7); and some others I won't point them out one by one here.
4. End-to-end genome assembly, do you mean Telomere-to-Telomere genome assembly.

Level of Interest

Please indicate how interesting you found the manuscript: Choose an item.

Quality of Written English

Please indicate the quality of language in the manuscript: Choose an item.

Declaration of Competing Interests

Please complete a declaration of competing interests, considering the following questions:

- Have you in the past five years received reimbursements, fees, funding, or salary from an organisation that may in any way gain or lose financially from the publication of this manuscript, either now or in the future?
- Do you hold any stocks or shares in an organisation that may in any way gain or lose financially from the publication of this manuscript, either now or in the future?
- Do you hold or are you currently applying for any patents relating to the content of the manuscript?
- Have you received reimbursements, fees, funding, or salary from an organization that holds or has applied for patents relating to the content of the manuscript?
- Do you have any other financial competing interests?
- Do you have any non-financial competing interests in relation to this paper?

If you can answer no to all of the above, write 'I declare that I have no competing interests' below. If your reply is yes to any, please give details below.

I declare that I have no competing interests

I agree to the open peer review policy of the journal. I understand that my name will be included on my report to the authors and, if the manuscript is accepted for publication, my named report including any attachments I upload will be posted on the website along with the authors' responses. I agree for my report to be made available under an Open Access Creative Commons CC-BY license (http://creativecommons.org/licenses/by/4.0/). I understand that any comments which I do not wish to be included in my named report can be included as confidential comments to the editors, which will not be published.

Choose an item.

To further support our reviewers, we have joined with Publons, where you can gain additional credit to further highlight your hard work (see: https://publons.com/journal/530/gigascience). On publication of this paper, your review will be automatically added to Publons, you can then choose whether or not to claim your Publons credit. I understand this statement.

Yes Choose an item.
